# Indonesian Artisanal and Small-Scale Gold Mining—A Narrative Literature Review

**DOI:** 10.3390/ijerph19073955

**Published:** 2022-03-26

**Authors:** Ami A. Meutia, Royke Lumowa, Masayuki Sakakibara

**Affiliations:** 1 Research Institute for Humanity and Nature, Kyoto 603-8047, Japan; sakaki@chikyu.ac.jp; 2 School of Environmental Sciences, Universitas Indonesia, Jakarta 10430, Indonesia; roykevanlumowa@gmail.com; 3 Department of Earth Science, Graduate School of Science and Engineering, Ehime University, Matsuyama 790-8577, Japan; 4 Faculty of Collaborative Regional Innovation, Ehime University, Matsuyama 790-8577, Japan

**Keywords:** Indonesian gold mining, ASGM, illegal mining, environmental and health impact

## Abstract

Indonesia is host to a long history of gold mining and is responsible for a significant contribution to world gold production. This is true not only with regard to large gold mining companies but also to small-scale mining groups comprised of people and enterprises that participate in the gold industry of Indonesia. More than two thousand gold mining locations exist in present day Indonesia. Artisanal and small-scale gold mining (ASGM) sites are spread out across thirty provinces in Indonesia, and have provided work opportunities and income for more than two million people. However, the majority of ASGM activities use rudimentary technologies that have serious impacts upon the environment, public health, and miners’ safety, which in turn generate socio-economic impacts for people residing around the mine sites. Moreover, many ASGMs are not licensed and operate illegally, meaning that they are immune to governmental regulation, and do not provide income to the regions and states via taxes. The possibility for more prudent management of ASGM operations could become a reality with the involvement and cooperation of all relevant parties, especially communities, local government, police, and NGOs.

## 1. Introduction

Community mining has been practiced throughout Indonesia for hundreds of years and is facing an even greater increase in activity in the present day. Indonesia is included in the top ten gold producers worldwide (with production in December 2020 alone reaching 130 tons) [1,2] and is host to large number of artisanal and small-scale gold mining (ASGMs) businesses and operational facilities. The definition offered by Wiriosudarmo (1999) in Prabawa (2020) [3] states that ASGM can be interpreted as any mining operation whose investors and participants are common people or belong to the local community themselves. Simultaneously, the Ministry of Environment and Forestry (KLHK) regulation defines ASGM as gold mining which is conducted by individual miners or small enterprises with limited capital investment and production. ASGM actors generally operate without a license and exploit marginal gold reserves located in remote areas with hard-to-reach access, such as protected or preserved forests, or even conservation areas. In some regions, however, the activities of ASGMs have also been found to be conducted in the middle of residential areas [4].

There are two types of ASGMs in Indonesia: one is licensed ASGM and the other is unlicensed ASGM. Most ASGM activity in Indonesia still operates illegally, because they do not possess a license permit from the government. Illegal ASGM is considered detrimental to the state because they are unlicensed, do not pay royalties, cannot be regulated, and contribute to damage to the environment, as well as adverse health impacts caused by the use of mercury.

The existence of ASGM also has an impact on micro-businesses’ development. ASGM activities are considered as occupying the informal sector, and as a means to provide job opportunities and sources of income [5,6,7,8], especially in light of the current COVID-19 pandemic [9,10]. At present, approximately two million people depend on ASGM activities for their living expenses, spread out across thousands ASGM locations in various regions of Indonesia [9]. The Ministry of Environment and Forestry states that such mining activities do not require special training. Many rural communities choose to make a living as miners or they combine farming and mining in order to subsidize their income. It is difficult for miners who earn money from processed gold taken from mining activities to move to other livelihoods. Such mining practices are becoming more widespread, strengthening in line with democratization and improvements in human rights awareness [11].

Most of the ASGMs in Indonesia use a traditional method utilizing mercury amalgamation in the gold extraction process. However, the use of toxic mercury results in severe environmental and health problems. Many alternatives to the harmful mercury amalgamation have already been studied [12].

Sustainable development in gold mining has received a great deal of attention in the last decade, including the legitimate Community Mining Area/WPR (*Wilayah Pertambangan Rakyat*) promoted by the government. However, implementing sustainable development in this industry has become a complex dilemma. Research on the potential for other types of business income is needed to ensure a legal, but sound livelihood for the miners. In order to minimize the amount of illegal ASGM activity, it is considered effective to organize mining actors into co-operatives and provide financial assistance. This would involve supporting them in switching to other types of businesses, whilst also providing environmental education with regard to the negative effects of mercury use and adapting appropriate technology to be used in its place.

In this article, we will set out a review of ASGM in Indonesia based on the papers that have been published in Indonesia and other countries regarding ASGM in Indonesia. In the last section, the author will propose that the problem of illegal mining and the dangers of mercury use that accompanies it can be resolved by the various parties concerned.

## 2. History, Location and Production

Indonesia has a very long history of gold mining (Table 1).

For quite some time, many local people have been engaged with gold mining in Indonesia in line with customary law; that being that rights related to gold mining are combined with the rights to possess land. Historically, inhabitants of the land offered payments to the chiefs, who provided permits to their communities to cultivate the land and develop forests. Gold mining rights took the same form as this. However, it was the 1899 Dutch East Indies Law that denied these customary mining rights (Table 1). This made it possible for European companies to operate by establishing a mining concession, proclaiming that the rights of landowners do not extend to the mineral reserves.

The customary idea that land right holders also have mining rights was continuously denied even after independence. For example, the 1967 Basic Mining Law stipulates that all underground reserves are controlled by the state (Table 1). ASGM managed by local communities, formed of small, mutually supported businesses that use simple tools for their livelihood, are stipulated to only gain rights to proceed with their activities upon being granted a license from the government. The dispute which has arisen between the customary idea that land right holders can mine and the national law that mining is allowed only when in possession of a government-issued license persists to the present day. Even now, a large number of ASGM actors are not licensed, but they believe that mining rights have been customarily granted. As such, this disagreement could be described as being at the heart of the widespread problem of illegal mining conducted by many ASGM actors.

We would argue that it is necessary to approach this problem by respecting the opinions of the inhabitants and recognize customary mining rights, rather than convicting ASGM actors as illegal miners. In fact, the 2009 Mineral and Coal Mining Act also states that mining activities conducted by communities in the area will be given priority as a Community Mining Area, as well as proposing to promote safe mining through cooperatives. In this way, what is needed is an approach that grants advantages to the local communities, paving the way for the regulated development of ASGM that would allow communities to make the best use of the reserves present in the area.

Gold mining takes place in almost all of the islands comprising Indonesia (Table 1). This is in part due to the existence of bountiful gold deposits along the western segment of the Sunda-Banda Neogene Arc, extending from Sumatra in the north, across Java, and continuing to Maluku in the east [30].

**Figure 1:**
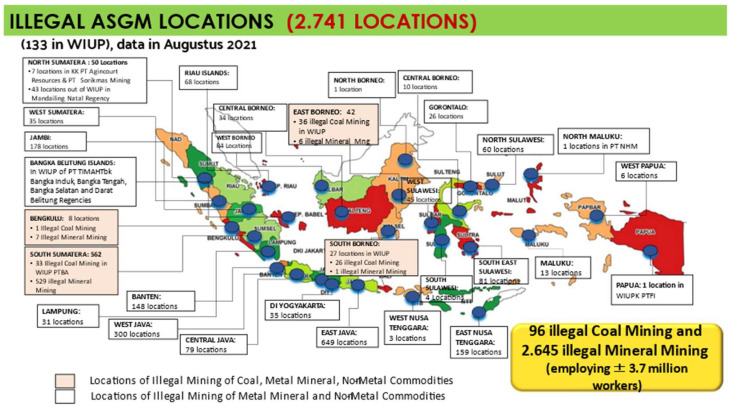
Locations of Illegal ASGM in Indonesia. Source: Directorate of Mineral and Coal of the Indonesian Ministry of Energy and Mineral Resources (2021).

ASGM activities have continued to increase quantitatively and qualitatively since the reforms for democratization and decentralization were introduced in 1998. According to data from the Directorate General of Mineral and Coal of the Indonesian Ministry of Energy and Mineral Resources [31], the number of illegal ASM (artisanal and small-scale Mining) including gold mining operations as of August 2021 amounts to 2645 locations spread across thirty provinces in Indonesia, with the number varying per province (Figure 1).

**Figure 2:**
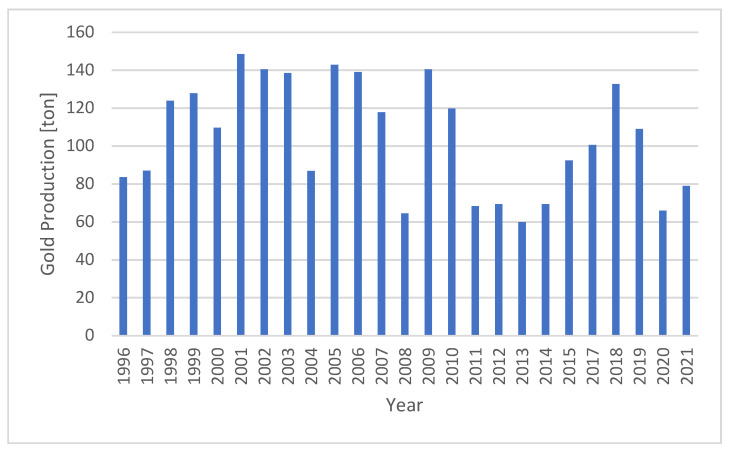
Indonesian Gold Production (Source: Statistics Indonesia).

**Figure 3:**
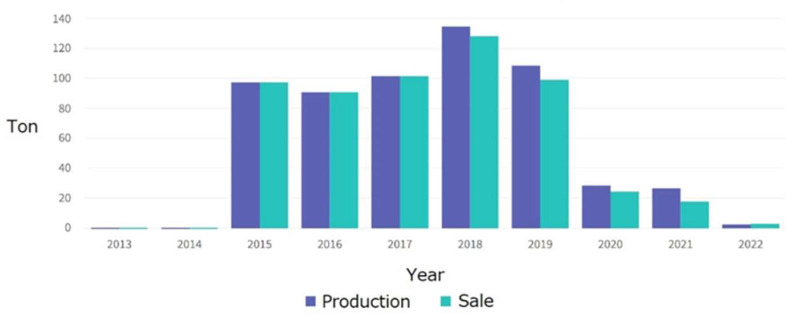
Gold production in Indonesia (Source: Ministry of Energy and Mineral Resources).

Table 2 shows data from companies that are still actively producing with their production, exports, and domestic consumption displayed. Figure 2 provides data of Indonesian gold production from Statistics Indonesia and Ministry of Energy and Mineral Resources (Figure 3), so that the graphs can complement each other, such as data in the year 2016 which is not available in Statistics Indonesia. Vice versa, data before 2015 can only be obtained from Statistics Indonesia.

The oldest producer in Java, ANTAM Co., was established as a state-owned enterprise in 1968 through the merger of several national projects and mining companies. Lately, there are also local governments that conduct gold mining activities together with private companies via share ownership, for example the South Tapanuli Regency Government and the North Sumatra Provincial Government in gold mining areas around Martabe, North Sumatra.

Information on the production costs of ASGM in 2012 and 2021 can be found in Table 3. It appears that the selling price at the location increased in line with the increase in world gold prices.

## 3. Illegal ASGM

**Figure 4:**
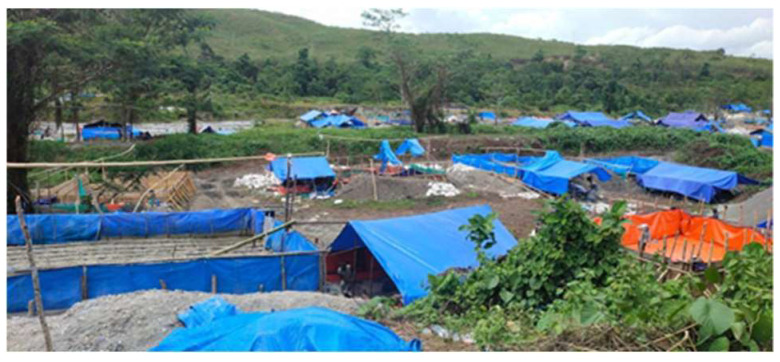
Illegal ASGM location in Buru Island, Maluku Province.

Unlicensed ASGMs, also known as PETIs in the Indonesian language, are operated by individuals or groups of people, sometimes conducted as a side business (Figure 4). One study shows, however, that due to increasing economic needs, illegal mining ventures are gradually being transformed from secondary businesses into main businesses [15]. One of the driving forces behind the increase in illegal ASGM is the excessive proliferation of gold mining companies competing in the same location [20,33].

Illegal ASGM is illegal according to Article 158 and Article 160 of the Law of The Republic of Indonesia number 4 of 2009 on Mineral and Coal Mining [28]. However, enforcement against illegal mining presents a significant dilemma for law enforcement officers. Raids and crackdowns on illegal gold mining have been conducted, but have not been shown to cause a deterrent effect for the actors. This is partly due to a lack of awareness surrounding the impact caused by illegal gold mining on the environment and human health. Moreover, the existence of illegal ASGM is directly related to the social and economic problems of the impoverished communities living around mining areas. In total, 77% of illegal ASGM miners reported that they experienced an increase in their general welfare thanks to illegal mining activities [6].

Throughout many generations, there has arisen a number of factors that have enabled the existence and increasing expansion of illegal ASGM practices. Firstly, the capital required to participate is relatively small. The businesses are conducted using simple (traditional) technologies, namely mercury or a combination process utilizing mercury and cyanide. Socio-economic factors such as limited employment opportunities, as well as the significant financial benefits offered by ASGM provide an attractive incentive for miners to engage in such illegal modes of business. These businesses have several characteristics in common, such as non-compliance with mining laws and regulations, lack of supervision frameworks, and resistance to law enforcement. Businesses often act under the knowledge that the process of obtaining a mining license is one fraught with complicated bureaucratic procedures, tending to incur high costs [6,15,34].

On the other hand, the unregulated development of illegal ASGM results in several negative consequences, such as: environmental degradation as a result of mercury use, changes in landscapes, forest/soil damage, water pollution, poor handling of mining waste due to the rudimentary skills used, issues related to health, mining accidents, high-interest illicit banking, monopolistic illicit trade, loss of state revenue, violation of the official taxation system, social vulnerability, disturbance of the community, legal abuse, unfavorable investment climates, security disturbances [15,35,36,37,38], and threats towards biodiversity [39,40]. Examples of such impacts, among others, have occurred in Mount Botak (Maluku), Lebak (Banten), Solok (West Sumatra), Poboya (Central Sulawesi), and in other regional locations in North Sulawesi, such as Ratatotok, Lanut, Lolayan, and Dumoga [5,41,42]. The large scale of illegal mining activity has been shown to pose serious threats to the health and socio-economic living conditions of communities situated around the mining sites [3,43,44]. Such threats have presented themselves continuously since the establishment of illegal ASGM sites.

According to Khotami (2020) [45], illegal ASGM also results in damage to food resources, as well as to breakdowns of established social order and discipline. However, the cooperative relationship between mining workers and investors is quite close, mutual trust, responsibility, tolerance, and prioritizing the attitude of kinship and mutual need for each other [46]. Additionally, in mining areas, located far from the reach of government supervision, the level of community income remains low and such conditions have been inherited from generation to generation due to mining activities that do not prioritize aspects of sustainable development [45,47].

The issues surrounding illegal mining is very complex in nature, so ideally any policy-based attempts to regulate illegal ASGM should be directed through a social approach that acknowledges the needs of the community, in conjunction with the need to enforce law. Extension programs or guidance administered to local communities are needed to overcome this problem. The level of control conducted by the authorities must be intensified so that the illegal miners (*gurandils* in local language), money brokers, owners, and investors (*cukongs* in local language) who operate in areas rich in gold mining do not run rampant. Illegal mining provides many benefits for the laborers, but even more so for the owners, as the exploitation of illegal miners offers significant profit opportunities for such secondary and tertiary parties. According to the accounts of several laborers, finances are often handled by unscrupulous persons, so that their mines can be operated securely, beyond the reach of the law. For each deposit, an allowance of two sacks of stone containing gold is allotted per hole. The value of each, however, does not necessarily depend on the gold content itself.

However, law enforcement and the forced closure of illegal ASGMs has had a significant impact on social security. According to residents of Lebak Regency, Banten, almost 99 percent of the people in their village work as an illegal miner. According to accounts in the community, since the closure of illegal mining facilities cases of motorcycle theft and other types of petty crime began to rise within the village. Apparently, this increase in crime is related to the population of illegal miners who have been left unemployed following the dismantling of unlawful sites.

From the aspect of law enforcement of illegal ASGM, this has become a serious dilemma, as it spans multidimensional factors such as social, economic, and legal aspects involving employment and poverty, as well as violations of law, so it is necessary to have policies that are able to accommodate such complex and wide-ranging problems [48,49]. Redi (2016) states that illegal mining law enforcement policy solutions should make policies based on cost and benefit analysis to ensure the achievement of the policy. Law enforcement officers and related officials must prioritize non-penal policies through monitoring and coaching small-scale miners towards a more legal form of mining [6].

Some more specific solutions include social outreach and education programs about the impact of illegal gold mining activities on the community, in conjunction with cooperation and coordination between related agencies and community support. The regulation of illegal gold mining can be conducted gradually and continuously, in tandem with strict law enforcement against illegal miners (workers and investors), prioritizing the implementation of deterrents towards local police officers suspected of being involved in extortion or bribery [7].

The police must also play an active role in implementing repressive, preemptive, and preventive measures towards the handling of illegal ASGM activity. However, it must also be recognized that law enforcement faces numerous constraints in this regard, held back by legislation, bureaucratic obstacles, lack of organizational infrastructure, as well as factors related to community legal culture [50].

As an example, by utilizing sufficient preventive and repressive measures, the Landak Police Department stated that the handling of illegal gold mining by the Landak Resort Police was met with some levels of success [43]. Similarly, the investigation into the criminal activity of illegal ASGM in South Solok Regency carried out by the Regional Police Special Criminal Investigation Directorate West Sumatra, based on the data obtained, was deemed to be moderately effective [51].

Based on one of the author’s experiences in collecting primary data as the head of the West Papua police region in 2016–2017, the head of the Maluku police region in 2018–2019, and the head of the North Sulawesi police region in 2020, it was found that there are four levels of capability designated to a police region in the handling of illegal mining (Table 4).

As shown in the table above, the effectiveness of a police region’s measures in enforcing against illegal AGSM can vary widely and is measured based on a number of interlinking factors.

Given the wide range of socio-economic factors that underlie it, one could argue that illegal ASGM activity will continue indefinitely. Millions of people depend on the labor it provides [52,53] and many regions rely on the large revenues from the mining business, including the state’s revenue generated from the acquisition of mineral resources. Illegal mining continues to operate in the Merangin, Sarolangun, Bungo, and Tebo mining areas in the Jambi Province, because the communities there view it as an expeditious method for generating profit. Such labor is easily accommodated by those who stand to benefit in the gold industry and is rendered secure by officers who are bribed to provide security and government officials who enforce regulations loosely [54]. As a result, more radical strategies are urgently needed to reorganize unlicensed mining activities, through improved management and environmentally friendly mining sites.

## 4. Impact of ASGM Activities

### 4.1. Mercury Problems

For ASGMs throughout various countries, the primary method by which gold is recovered during the mining process is via the use of mercury, usually in very high quantities. More than 1000 tons of mercury used in mining activities are released into the environment every year, and an estimated 10–19 million people are at risk from mercury exposure globally [55]. This problem is particularly exacerbated in many developing countries.

Almost all ASGMs in Indonesia use mercury to separate gold from ore in the amalgamation process. The mercury and gold precipitate to form a mercury–gold amalgam, which is then heated at high temperatures. Finally, the gold is extracted via evaporation of the mercury. Primary gold processing via the mercury method is less efficient, as it can only distill 10–40% of the contained gold [56]. Due to this, many miners conduct further processing using the cyanide method to extract the remaining gold from the initial processing waste. Very often in this way, miners in several areas process waste twice with cyanide, in order to maximize the produced quantity of gold. In several locations, it was noted that many miners believe that cyanide is capable of increasing the quantity of gold, whereas mercury increases its quality.

**Figure 5:**
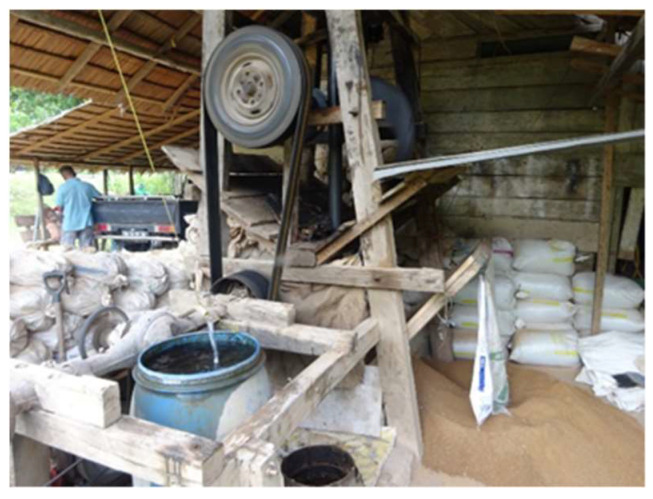
Process of grinding primary rock deposits (hard rock).

The traditional processing methods used by miners are generally only able to extract a small amount of gold with high levels of mercury and dust (Figure 5), causing environmental and health problems not only among the active miners involved in the processing, but also among the surrounding communities that are not actually involved in mining activities. For instance, mercury concentrations have been found in the hair of the heads of people living in the Tulabolo sub-watershed [57]. In addition to the elimination of mercury use, the treatment process could also benefit from improvement, so that its productivity can be increased and the processing waste produced can meet the accepted threshold value [30].

The United Nations Environmental Program (UNEP) 2018 states that global mercury emissions in 2015 amounted to 2220 tons, of which 49% came from Southeast Asia, 18% from South America and 16% from Africa. The small-scale gold mining industry is the largest contributor to mercury emissions with a total of 38% globally from 2010 to 2015 [48]. Indonesia is referred to by the United Nations as the third largest mercury emitter in the world, after China and India. Mercury pollution by the ASGM sector in Indonesia has increased significantly over the last two decades. For practical reasons, nearly 90% of small-scale gold mines in Indonesia still use mercury in their processing methods. Citing research by Ismawati (2013) in relation to mercury pollution’s effects on health, in Indonesia alone, around 195 tons of mercury were identified to be released into the environment per year. This amount represents 20% of global mercury emissions. Of this amount, about 57.5% of the mercury is released into the air, 15.5% into water, and 14% into soil or sediment [58].

Mercury pollution is exposed to humans via a variety of different routes. In the mining sector, it is poisoning due to direct exposure of workers to mercury. Outside of this, mercury contamination makes its way into the surroundings by spreading through rivers, agricultural damage, bioaccumulation of mercury in plants [59] which can produce harmful agricultural products (such as rice) [60], as well as mercury pollution in the ocean and mercury accumulation in fish [61] and seafood which is harmful to consumer health. Most ASGM sites in Indonesia possess no reliably safe method by which to dispose of mercury waste, resulting in miners generally depositing the waste into nearby rivers [26]. However, the main route of exposure is from the atmosphere. People can be exposed to mercury by breathing contaminated air produced during the amalgam smelting process, consuming contaminated food and through direct absorption via the skin. Mercury can cause digestive, respiratory, skin, and kidney problems, the effects of which can be very dangerous, even in minute quantities. At high doses, mercury can cause permanent damage to the brain and nervous system, kidneys [62], impaired fetal development [54,63], and lung damage [64].

In Indonesia, mercury has been designated as a hazardous and toxic substance (B3) by government regulations. Its use has been banned, and various measures have been taken to crack down on its employment in illegal mining. The Indonesian government signed the Minamata Convention on International Treaty in October 2013 and has ratified The Minamata Convention on Mercury into a domestic law by Law Number 11 of 2017 in September 2017 [65]. This commitment of the Indonesian government is carried out through the 2019 Presidential Regulation Number 21 in the National Action Plan for Mercury Reduction and Elimination (RAN-PPM), which aims to ban the use of mercury in ASGM as a National Priority Program and reduce/eliminate mercury contamination in an integrated and sustainable manner [66]. This is reinforced by the Regulation of the Minister of Energy and Mineral Resources of the Republic of Indonesia Number 16 of 2020 concerning Strategic Plan of the Ministry of Energy and Mineral Resources for 2020–2024 [67]. The Indonesian government has drawn up a national action plan for the reduction and elimination of mercury by 2030 [68]. This commitment was proven to be effective in Indonesia through real statistical examples. Until 2020, based on the RAN-PPM report [69], efforts to decrease the use of mercury in the ASGM sector resulted in a reduction of 10.45 tons of mercury. This was achieved not only through the curbing of ASGM that use mercury, but also by endeavors into the development of non-mercury-based gold processing methods.

Another important statistic to pay attention to is the increasing circulation and distribution of mercury sales in various regions. Scientists and environmental activists hope that global restrictions on mercury trade will raise mercury prices and reduce the mercury use and pollution involved in ASGM. However, in Indonesia, despite restrictions on global mercury trade, increased domestic supplies of mercury have conversely made mercury cheaper and more widely available. This phenomenon has the opposite effect of increasing environmental pollution and exposure to mercury by miners [58]. Consequentially, more far-reaching steps are needed by the Indonesian government to completely halt the illegal trade of mercury entering various regions. For that purpose, it has been argued that the government should play a more vital role in the reduction and use of mercury in small holder mining [70].

Amalgamation and cyanidation are the two main gold extraction methods that are currently in common use. However, these methods have a harmful impact on the environment and the health of miners and local people. Significant research on alternative gold processing outside the use of mercury has been carried out both on a global scale [71] and nationally within Indonesia [12], for example, the hydrometallurgical use of chemical solvents such as thiocyanate, thiourea, and thiosulphate. The advantages of the leaching technique using thiosulfate reagents include less environmental damage compared to commonly used methods and a faster dissolving process of gold compared to cyanide solution [72,73].

While there are other methods for extracting gold, the onus largely rests upon the miners. If the miners consider this method to be more efficient, cheap, and effective, they will naturally move from a dangerous method to using a more environmentally friendly and economical method. On the other hand, several problems arise in relation to alternative methods, such as the difficulty of changing the method because new equipment must be brought in to replace the equipment from the former method; an issue made all the more difficult due to the remoteness of the areas in which gold mining operates. Additionally, new methods are not easy for miners to learn and the necessary chemicals are not easily obtained by miners.

Alternative technologies to the traditional equipment in mining that uses mercury and other harmful materials is being sought by the government of Indonesia. Despite the fact that mercury is a material that is prohibited from being used in mining, the use of mercury is still ongoing even today, because switching to alternative technologies would incur large costs in procuring equipment that is difficult to carry out by illegal ASGM actors. Therefore, as a solution, the author proposes the concept of Willingness to Pay, so that the replacement to this alternative technology can be carried out immediately and the use of mercury in illegal ASGM can be stopped. Another idea is that more licenses will be issued by the government if simplification of the process to obtain a license is implemented on the condition that miners must use alternative mercury-free technologies. With the existence of TDCoP (transdisciplinary communities of practices) groups that carry out transformative learning [74] in good mining practice, awareness of the dangers of mercury and environmental safety and health, it is hoped that public awareness will increase.

In terms of solving mercury pollution, the author once again reiterates the use of the concept of WTP (Willingness to Pay). This can be successful due to the fact that it is not only miners and mining workers who suffer from the negative impacts of mercury use, but also people indirectly related to mining, due to the effects of environmental pollution. Taking that into account, the impact of mercury intoxication not only places an additional burden on the victim’s family, but also on the community in general. If we only rely on the concept of “those who pollute must pay for it”, the problem of removing mercury in mining will not be solved; it will persist as it has up until now. However, if the cost of eliminating the use of mercury in gold mining and the shift to mercury-free technologies is shared by all communities in a certain region, the problem of eliminating the use of mercury will be quickly resolved.

### 4.2. Environmental Impacts

Environmental pollution represents one of the most tangible adverse impacts of gold mining activities. Illegal gold mining in particular poses a remarkably higher threat to the environment, due to the increased danger to health and influence upon the occurrence of natural disasters it is directly responsible for. With regard to the latter, the effects of illegal gold mining have the potential to cause environmental damage in the long term, taking the form of negative changes to the landscape, landslides, and erosion, as well as water pollution in/around mining sites. A number of studies have reported upon the contribution of ASGM to land degradation [19,42,75], river water pollution [18,74,76,77], and soil/sediment pollution [18,25,26,36,57,78], whose conditions do not meet established quality standards. Indonesia Government Regulation 82/2001 for mercury in river water is 0.002 mg/L and in drinking water or in the water supply it is 0.001 mg/L.

**Figure 6:**
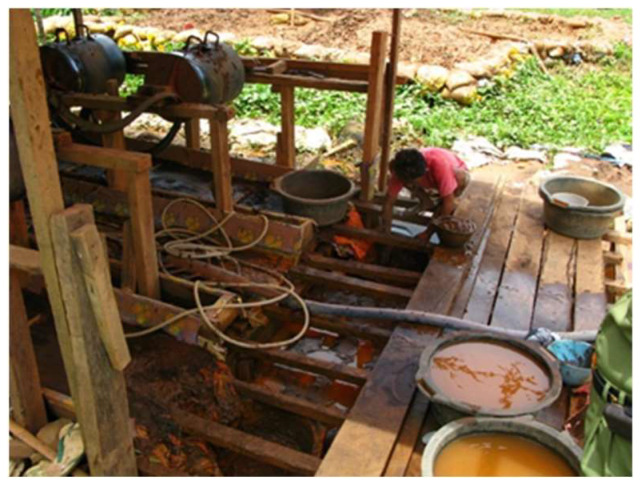
Gold processing sites.

Over time, the illegal mining that has been carried out traditionally (Figure 6) in Indonesia has resulted in damage to the agricultural land surrounding mine sites. The stagnation of water flows often leads to irrigation channels on agricultural land becoming a breeding ground for mosquitoes, whilst also polluting rivers and aquatic biota, ultimately altering the soil structure present around the mines [54].

As a result of mercury being distributed in the water sediments along Kayeli Bay, the aquatic ecosystem of Kayeli Bay has been contaminated with mercury as its bioconcentration (accumulation) was found in the leaves and rhizomes of the seagrass *Enhalus acoroides* [79]. Likewise, the concentration of mercury in sediments along the Tulabolo River, Gorontalo Province was deemed unsatisfactory by European Safety Standards and the water quality was found to be too close to the Government Regulation, PP82 threshold, 2001 [57]. The water and sediment mercury concentration of the Sekonyer River in the village of Aspai, Central Kalimantan was also found to have exceeded acceptable thresholds; a consequence of the boom in unlicensed gold mining which has occurred there since 1990. Additionally, the accumulation of mercury in samples of several types of fish and shrimp from the river exceeded acceptable EPA standards [78]. Similarly, the existence of gold mines along the Batanghari River, Dharmasraya Regency, West Sumatra has led to severe damage to the ecosystem around the river, causing the extinction of living things in the river and the cloudiness of the river water reaching levels that render it dangerous to consume [36].

A study which took place in the Batang Asai District, Sarolangun Regency, Jambi showed that the negative impacts of illegal gold mining activities resulted in not only a decrease in the quality of water, river, sediment, and soil but also to an increase in noise pollution, dust, forest conversion, river silting, the emergence of large holes, soil abrasion, and the disappearance of the meranti (*Shorea* sp.) and damar (*Agathis damara*) species of flora. Furthermore, a decline in the population of the semah fish (*Tor* sp.), once considered a common species to the area, was also measured [80]. Identical conditions related to the emergence of ground holes, loss of the semah fish population, forest vegetation decline, river silting, and cloudy river water were also noted in Muara Mensao Village, Jambi Province [47]. Mercury is not only found in fish and plants, livestock such as cattle in mining areas are also contaminated with mercury [81]. Finally, a high level of mercury contamination has been reported in various other ASGM communities throughout various provinces, such as in West Java [82], West Nusa Tenggara [62], Gorontalo [83], Southeast Sulawesi [84], and Buru Island, Mollucas [85].

As described above, environmental degradation from illegal gold mining exerts a significant influence upon river water pollution that extends to irrigation dams, causing the pollution of productive agricultural land and fisheries. Due to this, communities around the watershed often have difficulty accessing clean water for their daily needs and fishermen struggle to sustain their livelihoods. As such, the existence of illegal gold mining has led to numerous social conflicts. Likewise, incidents of landslides near mining sites have also added to the list of environmental damages and resulting social tensions [21,50].

The damage inflicted by ASGM operations has also been deemed a threat to world heritage geological sites along the Marupa and Kahayan Rivers in Indonesia, Central Kalimantan. Indonesia boasts a high level of biodiversity, with its tropical rainforests and unique geological characteristics, including the presence of forests in watersheds and mountains [40]. ASGM actors also frequently intrude and conduct activities within many national parks [20,39]. However, illegal gold mining activities greatly imperil the country’s biodiversity, as well as the world’s geological heritage as a whole.

From the various studies above, it is very clear that environmental safety is very important in mining. Mining sites containing hazardous chemicals such as mercury, etc. pose a risk to the environment. Mercury not only contaminates local mining areas, but can also be transported by rivers into the sea and can be carried by the wind to make its ways into plants and animals. According to several studies, traces of mercury have been detected not only in plants and animals around mining areas, but also in the bodies of fish, shellfish, and seaweed far from the mining site. Environmental safety is inseparably linked to public health. Even people who are not in direct contact with mining activities will also be affected if they eat fish or come into contact with objects which have been indirectly contaminated by mercury. Environmental safety is also an important concern for post-mining sites, because such land usually cannot be repurposed as agricultural, plantation, and fishery land for some time.

In order to combat the acidity introduced to water reserves, limestone can be added into reservoirs so that the pH of the water in the pond increases. In addition, for mining that has a clear post-operation plan, land surface arrangement and revegetation are conducted, such as the closure of pyrite material at a mining site with good soil material and planting vegetation on it so that the concentration of acid in the water is reduced.

### 4.3. Economic and Social Impacts

According to UNDP data, ASGM is an important source of income for Indonesia’s estimated 300,000 miners [86]. The gold procured by ASGM producers in rural areas can earn as much as 70% or more of the standard international price. With these considerations in mind, people living in remote areas often view small-scale gold mining as a way out of poverty [87]. The actors who participate in gold mining include agricultural and fishery workers who work part-time and require additional income in order to sustain their livelihoods. Meanwhile, for some communities, AGSM activity is the main source of income by which to support their daily lives.

In two provinces of Nusa Tenggara (NTB & NTT), especially in Sekotong Island-Lombok and Taliwang-Sumbawa, it is found that ASGM leads to an increase in economic activity, employment, income, and opportunities, meaning that they are considered as a form of positive social development [8]. However, over the generations, the lack of socio-economic benefits received by communities from illegal ASGM activities has been measured in the North Lebong District, Lebong Regency, Bengkulu Province [14]. In Kuantan Singingi, it has been noted that illegal gold mining tends to bring long-term benefits only to a limited number of actors, such as investors, and local police officers who accept bribes [7]. The findings in the province of East Nusa Tenggara and Sulawesi revealed however that existing mining sites there did not provide major long-term economic benefits to local communities [44].

In a similar nature, a study into the management of the gold mine in Bakan Village, Bolaang Mongondow Regency, North Sulawesi Province showed that the management of the gold mine brought about both positive and negative impacts. The positive impacts included the improvement of the miners’ immediate economic welfare, while the negative impacts included elements of danger related to health and the environment [5].

Ultimately, though migrant miners and those involved in the mining network benefited from AGSM activity, residents who are a part of the local community do not stand to profit from the mining operations, and instead suffer from the damage inflicted by destruction of their environment. In other words, any economic advantages earned from AGSM activity comes with the price of environmental degradation, as well as social vulnerability and injustice [21,88], including social security disturbances, and corrupt behavior [44]. It could therefore be argued that in order to avoid the negative impacts of AGSM, miners must operate only when in possession of a license. With a license, miners can work within strict safety standards that are maintained by work facility management [5].

The labor management system in illegal small-scale mining varies diversely and has no objective standards, as found in research related to small-scale gold mining in Pongkor, West Java. The absence of a defined labor system arrangement that is in accordance with labor standards puts mining workers at a major disadvantage. As a result, the laborers are not entitled to equal work and employment guarantees because there is no health insurance, safety guarantees, or regulations related to working hours [89].

From an economic point of view, mining was shown to improve the overall economy of Dharmasraya Regency, West Sumatera communities, especially those who were in possession of customary land rights along the banks of the river. On the other hand, the existence of mining facilities led to increased wealth disparity between the rich and the poor because only the owners of the capital and means of production stood to fully reap the benefits [36].

One phenomenon that is also commonly found is social conflict involving communities surrounding mining areas. Conflicts between communities and private companies began to occur in the Bogani Nani Wartabone National Park area, Bone Bolango Regency in Gorontalo Province after the government offered work contracts to private companies to mine gold. Even following the end of the Dutch occupation in 1940, traditional community gold mining activities are still illegal here, even though the land that has been owned by the community for generations will be passed on to their children and grandchildren [17].

Mining sites attract migrant miners from various regions, as seen in the example of the mining site of Talawaan-Tatelu, North Sulawesi Province, which is visited by miners from all over the province of North Sulawesi [78]. Disputes, or even fights between miners, as well as factions of miners involving inter-ethnic groups, almost always occur around mining sites. Such conditions lead to a poor sense of security and public order in the villages around the mining sites [51,54,88].

However, the illegal mining activity in Muara Mensao Village, Jambi Province has been shown to contribute significantly to the local economy. This is because rises in income can lead to positive impacts in the education sector. The education levels of the younger generation in the area revealed promising results [47].

In Perentak Village, Merangin Regency, Jambi Province, the existence of illegal gold mining conducted by Perentak villagers has had positive implications for household economies in the short term, but in the long term, environmental impacts caused by an increase in unlicensed gold mining has in turn brought negative implications for the average household economy. Any positive aspects brought about are offset by the fact that serious cooperation is needed between the community, government, non-governmental organizations, and the scientific community to prevent damage to natural resources in the future. Post-treatment of land following illegal gold mining activities is one method which could help to restore sustainable natural conditions [47]. Another would be the empowering of the traditional community by granting a platform for local wisdom to express itself, especially with regard to the economic disparity introduced to the community via AGSM. Accordingly, it is necessary to pay attention to the economic needs of the community, finding ways to provide benefits that grant advantages for the local villages [48].

ASGM is one of the economic activities that has been hit hardest by the COVID-19 pandemic. However, the COVID-19 outbreak can be used as an opportunity by which to reconfigure public opinion towards ASGM activities, instead allowing us to view it as a safety net during economic crises, that can act as a fallback for local economies that have been unable to find alternative methods of income [9].

In conclusion, ASGM needs to be recognized as a formal economic sector with solid legal foundations, in order to maximize the benefits and minimize adverse impacts brought about by the sector. An approach which integrates the idea of sustainable mining is one method by which to move forward, counteracting the current exploitation of natural resources and unsustainable resource management [7].

### 4.4. Impact on Health and Education

**Figure 7:**
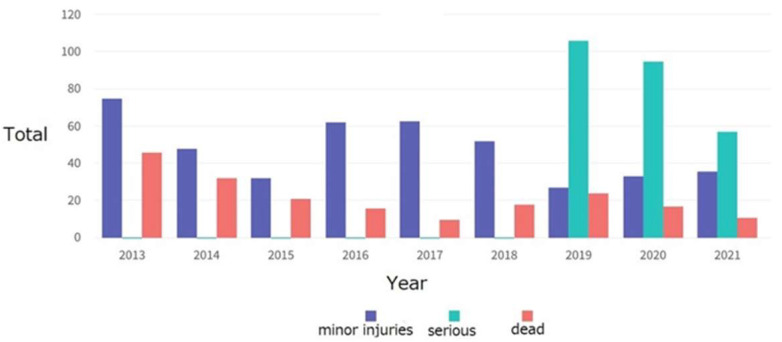
Total number of accidents in mining companies including gold mining (Source: Ministry of Energy and Mineral Resources).

Illegal ASGM is very harmful to the safety and health of workers. Every year, several accidents occur at illegal ASGM sites. Figure 7 shows total data from registered mining companies for all types of mining. As such, it cannot reveal data related to illegal ASGM, only displaying statistics related to legally operated mining.

Table 5 shows that accidents that occur in illegal ASGM are very frequent every year, caused by landslides, oxygen deprivation, and toxic gas inhalation. Therefore, it is very important to legalize ASGM by giving illegal miners licenses so that they can be taught how to conduct safe mining practice and minimize mining accidents in TDCoP groups through transformative learning.

Buru Regency is one of the main areas in Maluku which experience health problems as a direct result of the use of mercury and cyanide in the amalgamation process of traditional gold mining [26].

Illegal mining has caused many cases of health deterioration [83,84,90] and disease in the community [80], as well as amongst the miners themselves, stemming from the identification of mercury traces in the miners’ bodies [91,92,93]. This is revealed by the research that was conducted in Ratatotok, Southeast Minahasa Regency, North Sulawesi Province in 2019 [58] and the accounts of a number of residents living around Mount Botak mining area on Buru Island [85]. In North Lebong District, Lebong Regency, Bengkulu Province, the community felt that illegal ASGM practices caused nearly 57% of all health issues, such as coughing, lung problems, and tuberculosis [14]. Moreover, the risk of soil and plant contamination through mining activities has led to high concentrations of mercury building up in the bodies of local residents within a short period of time [8].

**Figure 8:**
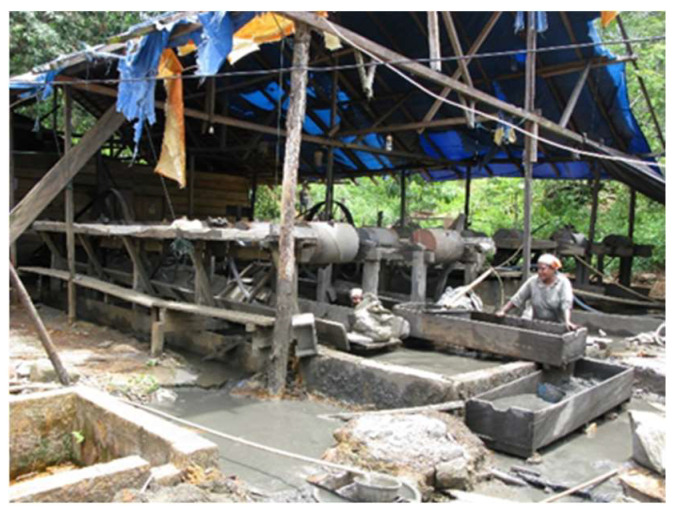
Female worker at an illegal ASGM, carrying out gold processing work without gloves.

The impact of mining activities is serious, especially in children and women, the most vulnerable, who participate in mining or live around the mining areas of Merangin, Sarolangun, Bungo and Tebo, Jambi Province. As illustrated in Figure 8, female laborers in the mines working without using gloves is a common sight (Figure 8). The immediate influence on health problems includes impaired growth and development of children living in locations around the mine, with long-term impacts including the threat of permanent disability and malignancy to children and the overall community [54,63].

Health problems and diseases that arise in gold miners come in the form of chronic and acute diseases. Chronic diseases caused by mercury intoxication in gold miners include the occurrence of liver dysfunction, decreased leukocytes, paralysis of limbs, numbness, and tremors (Parkinson’s disease). Tremors is a condition where the hands and feet are always shaking, while the facial muscles and lips often move unconsciously. Other health problems that arise include a lack of passion for activities (depression), difficulty sleeping, emotional turbulence, poor memory, cramps during weather conditions, cold, and feelings of anxiety. While the acute diseases that arise are acute poisoning, diarrhea, upper respiratory tract infections, eye diseases, vertigo, miscarriage, and skin diseases.

In the case of childbirth, there are inhibitors to fetal development because organic mercury from the methyl mercury form can enter the placenta and inhibit fetal development in women who are pregnant. This can cause birth defects in the baby, damage DNA, and interfere with blood flow to the brain, causing damage to brain tissue.

Table 6 shows symptoms of miners/inhabitants caused by mercury intoxication. Data regarding children born under the influence of mercury intoxication are the results of reports conducted by BaliFokus and Medicuss Foundation in Cisitu (Lebak Regency, Banten Province), Bombana Regency (Southeast Sulawesi Province), and Sekotong (West Lombok Regency, West Nusa Tenggara Province). They found several severe mercury intoxication suspects in adults and children. The studies were supplemented by measuring mercury levels in hair or blood. The results showed that in some samples the internal mercury levels exceeded the permissible standard values. There are some photos of children suffering from synostosis, seizures, hypersalivation, short size hand, kriptorkismus, labiognatopalatoschizis, congenital talipes, cataract juvenile, deafness, anokuli, and high myopia [94]. Unfortunately, it is quite difficult to obtain data from the local health office, as reports and previous studies are often incomplete and accurate.

Education of children is often neglected at illegal mining sites, and there are often no schools or educational facilities in many mining districts. In addition, the presence of sex workers who make a living around mining sites holds the potential for causing a myriad of other social problems. Overall, living conditions and general wellbeing of residents around mining areas are often poor, meaning that a comprehensive approach would be required to tackle the wide variety of problems being faced there.

One form of good mining practice is an environmental education and the teaching of how to make mining tunnels that are safe from landslides, oxygen deprivation, and toxic gases. Miners and mining workers require the use of personal protective equipment (PPE) such as rubber gloves, masks, shoes, and helmets. Meanwhile, for owners/funders of illegal mining, the use of PPE compounds the difficulty, because there are many cases of gold loss both in excavation and processing which are actually hidden in PPE equipment by workers (interview result with owner). The use of alternative technologies prevents the community from being affected by mercury intoxication. However, during the transition period, while they are still using these hazardous chemicals, it is necessary to conduct periodic health checks for miners. This routine health test should be carried out by the Community Health Center (Puskesmas) or Health Office in the province. Our project has established the Health Village (*Kampung Tangguh Kesehatan*, KTK) in the East Suwawa, Gorontalo Province in collaboration with the local government in planning to carry out health checks for the miners. Small groups of TDCoPs can increase communities’ awareness by teaching more about the dangers of mining using mercury.

## 5. Government Role and Policy/Customary Law Involvement

### 5.1. Government Role and Policy

Natural resource management is inseparable from the issue of licensing illegal mining activities. Every mining actor, both individual and enterprise alike, is required to obtain a license as regulated in Regulation Number 4 of 2009 [28] regarding Mineral Mining in conjunction with Government Regulation Number 23 of 2010 [100] about the Implementation of Mineral Mining. The rise of unlicensed gold mining makes it difficult for the government to supervise. All existing regulations and laws work together to regulate the actors of gold mining activities, in a manner which seeks to prevent permanent damage to the environment. The combined functions of law enforcement are based on the rules contained in Law Number 4 of 2009 with regard to Mineral and Coal Mining, UU Number 32 of 2009 [101] pertaining to Environmental Protection and Management, Government Regulation Number 23 of 2010 [100] about the Implementation of Mineral and Coal Mining Business Activities, and Regulation of the State Minister for the Environment Number 23 of 2008 [102] concerning Technical Guidelines for the Prevention of Pollution and/or Environmental Damage Due to Community Gold Mining. Law enforcement is carried out against parties involved in illegal gold mining using a number of both preventive and repressive measures. However, when it comes to the implementation of the above policies, the laws are very much subject to violation, especially regarding licensing issues.

Following the enactment of Law Number 23 of 2014 [103] concerning Regional Government, effective from October 2016, the designated roles and policies to be enacted by local governments became far more palpable. Law Number 23 of 2014 concerning Regional Governments emphasizes that regents and mayors are no longer authorized to assign mining business areas and mining business permits to enterprises. That authority now belongs only to the governor and to the central government. Prior to 2 October 2016, local governments had the authority to prohibit and control mining activities. However, after the new law was instated, the local government was relinquished of any authority over mining activities in the area, which was now in the hands of the provincial government. In reality however, far from being more regulated, mining in Indonesia is actually becoming increasingly less controlled, and is instead experiencing rapid expansion. The latest Law Number 3 of 2020 [29] introduced a shift in authority from the provinces to the central government (Table 1).

Environmental damage and mercury waste pose a severe threat to the people of Prabu Village, Central Lombok Regency, West Nusa Tenggara. The government closed this village’s gold mining site, resulting in conflict between the government and the mining communities [27]. The closure of this mine was ultimately ineffective, since the residents continued to carry out mining activities in secret.

Another definitive example can be found in the case of illegal gold mining in Banyumas, which has also been subject to numerous issues in relation to governmental intervention. The majority of mining that takes place in Banyumas is of a traditional, and consequentially illegal nature. In this manner, illegal mining could be said to be a form of vertical conflict between the community and the local government, causing dilemmas for the local government in the prohibition and control of mining operations [88,104,105,106]. The law enforcement of illegal gold mining in Banyumas Regency is implemented by forcefully stopping mining activities in the field, sealing mining sites and seizing goods related to mining activities [105]. In order to guarantee its success, strong cooperation between the government, the community, and the private sector must continue and should be executed under strict supervision.

Furthermore, support from non-governmental organizations (NGOs) is needed to handle the ongoing crisis of unlicensed gold mining. The role of NGOs as an intermediate actor allows them to act as a balancing force, offering greater empowerment to the unlicensed miners [85,107].

Observing how institutions deal with illegal gold mining in Kuantan Singingi Regency, Riau Province, it has been shown that the leadership role holds little sway, both formally and informally, in supervising the activities conducted by capital owners and mining actors. In addition, there were no regional regulations implemented that could act as a legal umbrella over community mining. Limited resources in terms of costs and facilities, and the remote distance of mining sites make it difficult for police to enforce control. Cooperation is needed in institutional development between provincial and district governments, the Regional House of Representatives and the community in overcoming the problem of illegal gold mining in Kuantan Singingi Regency [45].

Actions taken by the government up until now include the closing of many mining sites operated by illegal ASGM, in collaboration between the local government and the local police. Although the closure of mining sites is not an easy task because of resistance from the mining community, limited government officials or police, locations deep in the jungle or on top of mountains that make it difficult due to limited access, and the presence of people who protect the existence of illegal mining activities.

The government has a responsibility to take anticipatory steps in preventing health problems caused by mercury and other heavy metals in the human body, especially children living in proximity to illegal mining areas. It is important to advocate for policy makers to construct policies that protect people’s health and prioritize environmental conservation [54].

The problem of stopping illegal mining in Indonesia does not only concern one governmental department. Although there are already clear regulations, it seems that changing regulations and shifting powers would allow for various opportunities. For the government authorities, the authors believe that cooperation between relevant departments, police, and between local and central governments is the best solution to stop illegal mining. Meanwhile, from the community point of view, they need certainty for livelihoods, security and justice in obtaining natural resources around their area. The viewpoint shared amongst illegal miners is that they do not want to be spectators in their area, where their area’s natural resources are being exploited by companies, most of which are not from their area or even from abroad (interview result). Due to this, illegally operated mining can commonly be found being conducted around/adjacent to large-scale official (company) mining areas, resulting in conflicts with the mining business license holders [15].

The current reality is that many traditional mines are located in mining company locations. Therefore, it is necessary to make additional rules that accommodate both in the same location.

### 5.2. Customary Law

Under the Indonesian Mining Law, unlicensed mining activities are considered criminal acts regulated by Law Number 4 of 2009 concerning Mineral and Coal Mining. In spite of formal legal attempts to curb unlicensed mining, ASGM activities operating beyond the reach of the law are still ongoing. There has in the past been cases where instances of unlicensed gold mining were resolved using alternative, community-based legal instruments, in the form of customary sanctions in Kualan Hulu Village, Simpakng Hulu District, Ketapang Regency [108]. These customary legal sanctions against illegal mining actors in Kualan Hulu Village were considered effective in instilling a sense of justice, whilst achieving the objectives of the applicable law. The application of customary law in implementing sanctions against illegal mining actors is something which could also be adopted into the governmental mining law system in Indonesia [108].

Perhaps one of the reasons why customary law could function effectively in combatting ASGM, is related to the trust and social capital that community leaders command, in stark contrast to the lack of trust garnered towards government officials. Resistance can be established from a bottom-up perspective, allowing for new internal norms to be instilled, that can be adhered to by the community [41]. The disparity in trust between community and government was made apparent in the granting of a mining contract of work permit in the Central Sulawesi Grand Forest Park to a private company. This resulted in clashes of perspective between the government and the community which ended up with the development of an illegal mining site managed by the community.

In several villages in Indonesia, customary law (*adat*) plays a role in governing their communities. Traditional customary institutions were previously able to regulate how to manage the environment. Several villages have proven to be able to conserve the environment by prioritizing the functions of customary institutions. In the illegal gold mining areas of Kuantan Singingi Regency, the function of traditional customary institutions in preventing environmental damage, however, has gradually weakened. Therefore, the revitalization of customary and legal institutions is an urgent task in the battle for environmental protection [109].

## 6. Prudent Management of ASGM

As described above, there are two major impacts of illegal mining on the community, positive impacts such as increasing community economic welfare and negative impacts, namely environmental damage and decreased health quality due to mercury intoxication. Meanwhile, the impact to the government is the loss of income tax from mining activities and environmental damage, the repair of which requires government expenses.

One way to tackle these effects would be through prudent management. Prudent management of ASGM is an alternative approach which should be taken immediately in order to prevent and reduce severe damages to the environment and food resources. This must be realized together through a strong and integrated collaboration between the government, the community, and the private sector. Furthermore, a strict, consistent and non-discriminatory form of law enforcement should take place in the handling of ASGM [36]. Such enforcement must intimately involve the relevant government institutions and policing authorities [7,105]. This is due to the wide range of participating actors and the complex socio-economic processes and structures which ASGM involves [109]. The inhibiting factors faced in constructing a sustainable form of mining development are primarily a result of the lack of legal awareness in mining communities, as well as the rampant corruption taking place on both an individual level [43,105] and an organizational level, especially with regard to the behavior of officers [44] who accept illegal levies.

Indeed, such incidents must be stopped and sensible management of mining must be implemented, so that these conditions in which severe environmental damage and the overexploitation of natural resources are permitted to occur do not persist. Mining management begins with the regulation of unlicensed miners, which deters them from engaging in activities at the sites and allows for the closure of the unlawful mining sites [3].

The formalization of ASGM is a demand that must be followed up immediately [9,49] by the relevant agencies. This change in system or regulation can bridge the needs of community mining with the government that has the authority to issue and supervise mining permits [9,49,109]. Such efforts, on the one hand, would allow miners to obtain permits through cooperatives or associations. In addition, a strict environmental management on the safety and health of miners, as well as environmental quality around the mine site can also be maintained. However, the processes of legalizing community mining must account for strict environmental considerations and be supervised by impartial and fair institutions [8,9,48]. Such processes must also take into consideration the perspectives of miners and local communities [44,49] in a balanced way.

Therefore, in the case of legalizing community mining, the customary rights of indigenous miners must be recognized. Additionally, companies that wish to operate in a community mining location must be able to co-exist with community miners who have worked there for decades.

At the community level, TDCoPs (transdisciplinary communities of practices) of small groups can be used so that the community’s perspective on good mining practice and health can be changed through transformative learning. TDCoPs can be applied throughout various layers of society. In the Suwawa Timur region, in collaboration with the local government, there is a Health Village (a combination of several TDCoPs) which aims to raise public awareness of good mining practices, mercury impacts, environmental concerns, and community safety and health. At the Health Village, the community can learn environmental education, especially concerning mercury and mining. This project is available for children, elementary and junior high school students, as well as women (Pateda, personal communication) [110].

## 7. Discussion

The ASGM problem in Indonesia is already an exceedingly complex one. It is however becoming further complicated and distinct compared to the activities taking place in other countries, because illegal ASGM in Indonesia has specific problems related to history and makes it difficult for the government to control through regulations alone. Traditional people’s mining that has operated since hundreds of years ago is not recognized by the government and is considered illegal, with regulations made later and applied retroactively. However, people who have been mining for generations or have customary rights to their land have different opinions from the government. According to the author’s opinion, mining customary rights of local people should be respected, and mining licenses (IPR) should be granted to those who were actively mining before the regulations were made, and exercised their customary rights. Miners should be organized as cooperatives that function via rules which are compatible with government regulations, and must follow the rules and regulations made by the government. The neglect of local people’s rights is the most important problem that is protracting the issue of illegal mining in Indonesia. Miners working via cooperatives would be able to employ alternative technologies that avoid contamination from mercury. Simplifying the process of granting mining licenses would actually be very beneficial for the government, because the government can find out the exact number of miners who operate illegally, can urge the miners to protect the environment and health, as well as gain significant revenue in the form of taxes. For mining workers with low education, it is necessary to provide training to find other forms of livelihood through transformative learning in a TDCoP group.

Even though illegal ASGM activity has been prohibited amidst a flurry of changing regulations, community actors continue to conduct illegal ASGM. If these circumstances do not change, the environment will suffer from further deterioration, and in the end, no parties will benefit. The economic situation of the community around mining areas is not improving, whilst at the same time the government cannot control the illegal ASGM.

The government cannot obtain tax revenue from illegal ASGM, since illegal ASGM actors are not officially registered as an individual, cooperative, or company. The mining sector is one of the biggest non-taxed sources of income for Indonesia. It is estimated that the potential loss of state revenue in 2019 is at least USD 908,544,000 per year [111]. In addition, the post-mining environmental damage that remains after gold has been fully extracted from the land, without any rejuvenation measures enacted by illegal ASGM actors, will end up as the burden of the local government. The conditions of the people who are sick and suffer due to mercury poisoning will also become a burden to the community and will indirectly result in losses to the local government. If the tax from mining can be procured, it can be used in a sustainable manner for regional development and environmental conservation.

The above Table 7 shows a comparison between illegal ASGM and mining companies in Indonesia. It is shown that illegal ASGM results in more losses than benefits. Therefore, to replace current mining practices, legalization of ASGM is an urgent need. The closure of illegal ASGM sites alone is not enough because it will eliminate people’s livelihoods. Thus, the local government should cooperate with the central government to find the best way to simplify the process of ASGM legalization. ASGM legalization will introduce multiple benefits, both socio-economic and environmental. However, the reality is that not all illegal ASGM locations can be legalized, such as activity taking place in national parks or on land owned by mining companies. For this reason, it is necessary to provide alternative livelihood solutions for the people affected by the illegal mining closures. One way is by transformative learning through TDCoP (transdisciplinary communities of practices).

Currently, what the government is doing to control illegal mining is the closure of ASGM illegal mining sites and the requirement of anyone who wants to partake in mining as ASGM to obtain a license directly from the central government (new law Number 3 of 2020). Previously, the granting of licenses was the authority of provincial governments. The function of local governments now is to submit applications for a Community Mining Area (WPR) to the central government. After a Community Mining Area is established, the individual or cooperative can apply for a mining license (IPR). Many people are reluctant to apply for a mining license because the community mining location does not contain significant gold (information directly from miners). Especially if there is a Mining Business Permit Area (WIUP) owned by a mining company in that area (which actually came after the community started mining). The company is granted the area by the central government. Thus, the Community Mining Area cannot take place in the Mining Business Permit Area. As a result, the community ends up with land that is less suitable for gold mining and of no interest to miners. Therefore, it is necessary to make additional rules to allow the community and mining companies to coexist in adjoining locations. There are also people who are reluctant to apply for a license because it is a complicated, expensive, and time-consuming process to obtain a mining license. Therefore, even though the number of Community Mining Areas is large in quantity, the number of license holders is still low. To date, only 100 licenses have been issued throughout the 3329 Community Mining Areas. In fact, there has been no new licenses issued after Law No. 3 2020 (personal communication) [112]. Simplification of the time-consuming, difficult, and costly licensing process will lead to an increase in legally operated ASGM.

Another problem, however, is that mining companies who do obtain permits from the government often expel traditional (illegal) miners from their regular working sites. This has led to conflict between companies and local communities. Therefore, there must be a solution in the form of new regulation or an intermediary regulation that accommodates the presence of traditional miners in locations adjacent to license-operated mining companies.

The next step is to charge the cost of eliminating the use of mercury for all communities in a region. The benefits of improving the environment, public health, and removing mercury are enormous. If the costs for these benefits are shared, it will relieve the miners so that they will not use mercury in traditional people’s mining. For that purpose, we should apply a methodology of evaluation of environmental hazard by ASGM and mercury contamination. For example, with the concept of WTP (Willingness to Pay), we can evaluate the negative impacts on the environmental ecosystem in a particular region, and the local government can charge a cost, in order to offer alternative ways of mining, such as applying intermediate technology to avoid mercury contamination, offering alternative sources of livelihood for the miners, managing the cooperatives for ASGM small miners, environmental education and so on.

Above all, resource utilization should use the best possible recovery approach. In line with this, we offer the following suggestions:

a. People’s customary rights to mine should be respected. Though difficult, time-consuming, and costly, simplifying the legalization process is considered to be one possible way. Another would be the making of additional or complementary regulations, so that traditional people who have been mining in a location for a long time are not expelled due to the arrival of companies that have mining permits in that area. Alternatively, compensation money could also be offered.

b. Dialogue with the community and raising public awareness of good mining practice without mercury, improved safety and health through transformative learning by creating small TDCoP (transdisciplinary communities of practices) groups.

c. Alternative and appropriate technologies should be supplied to avoid the use of mercury. Miners would be organized in the form of cooperatives where such alternative technologies would be readily supplied, as well as forward-thinking measures such as credit, environmental education, and the offering of alternative livelihoods.

d. It is also necessary to introduce other alternative livelihoods for the community that are valuable and in line with sustainable development through transformative learning.

e. In order to cover the cost of operating the cooperatives, the author proposes to apply the concept of Willingness to Pay (WTP). In this way, people who live in provinces or regencies that suffer from the environmental damages caused by mercury use via illegal ASGM activities could share the cost to eliminate the use of mercury in illegal gold mining. This method has merits, as ultimately, almost all residents in the province/regency will face the negative impact caused by environmental degradation. The funds collected must be managed prudently by the government and be used to replace traditional equipment using mercury with alternative technology, offer training in the use of this new equipment and how to conduct good mining practices, restoration of post mining areas, environmental conservation, and routine health checks for miners, so as to achieve sustainable development. This can only be achieved if the funds are treated according to their purpose.

## 8. Summaries

Based on the review of the article above, it can be summarized that:

1. Artisanal and small-scale gold mining (ASGM) in Indonesia is conducted in more than two thousand locations in thirty provinces and most of them operate without a license. The mining activities are practiced using simple technologies that employ mercury and cyanide, which is not environmentally friendly.
2. It is an urgent task for the government to regulate ASGMs immediately, in order to stop further environmental degradation, overexploitation, impairment of environmental health, and reduction in the socio-economic wellbeing of surrounding communities.
3. Wise management of ASGM can only begin by cracking down on miners who work unlicensed and by closing illegal mining sites, followed by the legalization of Community Mining Areas (WPR) where miners and their associations can apply for Community Mining Licenses (IPR) in order to work legally.
4. The formalization of ASGM is necessary and should be enacted immediately, in a way which bridges the needs of actors in the mining industry and the priorities of the government that has the authority to issue licenses and supervise activities. The procedures of legalizing community mining should be framed by strict environmental considerations and supervised by impartial institutions, whilst also including the participation of local people, considering the perspective of local knowledge and traditions of miners and communities.
5. The community must be educated to increase their awareness in terms of good mining practice, mining safety and health, as well as the dangers of mercury and its consequences through transformative learning in TDCOP (transdisciplinary communities of practices). The community must also be provided with opportunities for other forms of livelihood that are more valuable and sustainable than gold mining—an unsustainable industry which will eventually come to an end in the future.
6. By using the concept of WTP (Willingness to Pay), the negative impacts on the environmental ecosystem in a particular region can be reduced and alternative technologies without mercury can be more widely disseminated.

## Figures and Tables

**Table 1:** History of Active Mining and Law in Indonesia.

Year	Location and mining operation/activity	Law
8th century AD (Hindu Period)	* West Kalimantan [13,14] * Sumatera * Java	
1669–1928	* West Sumatera: Salido Ketek	-
1760–1880	* West Kalimantan: Landak (China District) by Chinese immigrant [15]	-
1850–1899	* North Sulawesi: Bukit Mesel (1850) *Bengkulu: Lebong (1896) by Lebong Goud Syndicaat	* 1899: A mining law called the Indische Mijnwet Staatsblad was issued [16].
1910–1939	* Bengkulu: Simau (1910), Salida (1914), Lebong Simpang (1921) and Tambang Sawah (1923) [17,18] * West Sumatera: Tambang Manggani (1913), Belimbing, Gunung Arum (1935), Muarasipongi (1936)	-
1940–1941	* West Java: Cikotok (1940) * Riau: Logas, Kuantan Singingi [19] * North Sulawesi: Tapaibekin & Ratatotok (1940) [20] * Aceh: Meulaboh (1941)	-
1950–1959	Most of the people’s mining is active: * Bengkulu [21] * Kalimantan * North Sulawesi Mining company (State Company Antam): * South Banten: Cikotok * Riau: Logas	* 1959: Law Number 10 of 1959 was issued regarding the cancellation of mining rights.
1960–1967	-	* 1960: Indische Mijnwet was replaced by Government Regulation in Lieu of Law Number 37 of 1960 concerning Mining [22]. * 1967: the Foreign Capital Investment Law was introduced, together with the New Mining Law Number 11.
1972–1989	* Papua: Ertsberg, (1972 & 1980), Grasberg (1989) * Banten: Cirotan, (1978) * Central Kalimantan Ampalit (1988)	-
1991–1998	* North Maluku: Lerokis (1991), Kali Kuning (1994) * East Kalimantan: Kelian (1992) * Central Kalimantan: Gunung Muro (1994), Mirah (1995) * West Java: Pongkor (1994) * North Sulawesi: Messel (1995) * North Sumatera: Martabe (1997) * NTB: Batu Hijau (1998) [23]	* 1992: Government Regulation Number 79 of 1992 concerning mining permits from the Minister of Mines was issued [24].
2000–2020	* North Maluku: Gosowong (2005), Buru Island [25,26] * East Java: Tambang Tujuh Bukit, Banyuwangi (2017). * West Nusa Tenggara: Prabu Village, Lombok Regency (2011) [27]	* 2001 & 2004: Government Regulation Number 75 of 2001 and Law Number 32 of 2004 on local government explaining the mining management authority is on the local government. * 2009: Law number 4 of 2009 was issued regarding mining [28]. * 2014: The enactment of Law number 23 of 2014 concerning the revocation of mining management authority at the district/city level. * 2020: Law number 3 of 2020 concerning the transfer of mining management authority from the provincial government to the central government [29].

**Table 2:** Top 15 Gold Companies.

Company Name	Production [ton]	Export [ton]	Domestic [ton]
Antam Co. (UBPP Logam Mulia)	44.13	17.60	13.70
Freeport Indonesia	28.01	11.63	19.51
Agincourt Resources	12.17	11.93	0.00
Tambang Tondano Nusajaya	6.8	7.03	0.00
Nusa Halmahera Minerals	5.1	5.55	0.00
J Resources Bolaang Mongondow	2.6	2.78	0.00
Indo Muro Kencana	1.92	1.87	0.00
Amman Mineral Nusa Tenggara	1.73	0.83	1.17
Bumi Suksesindo	1.56	1.56	0.00
Antam Co. (UBPE Pongkor)	1.42	1.05	0.00
Meares Soputan Mining	1.33	1.34	0.00
Natarang Mining	0.9	0.74	0.00
Kasongan Bumi Kencana	0.86	0.86	0.00
Sago Prima Pratama	0.49	0.49	0.00
Sultan Rafli Mandiri	0.01	0.00	0.00

**Table 3:** Gold Cost Production of ASGM.

Year	Cost Production [Rp./gram]	Gold Price [Rp./gram]	Profit [Rp./gram]	1 US Dollar (December)	Reference
2012	78,400	250,000	171,600	9670	Anang Suherman [32]
2021	100,000	500,000	400,000	14,266	Prabawa [3]

**Table 4:** Designated levels granted to police regions in their combat against illegal ASGM.

Level	Leniency	Bribery	Enforcement Success	Future Outlook
Level 1	Permits illegal ASGM activity	Accepts illegal levies	N/A	Unchanged
Level 2	Does not permit illegal ASGM activity	Does not accept illegal levies	Poor	Little change
Level 3	Actively enforces against illegal ASGM activity	Does not accept illegal levies	Successful	Illegal sites closed, but no steps towards legalization of sites
Level 4	Actively enforces against illegal ASGM activity	Does not accept illegal levies	Successful	Groundwork laid for the transformation of illegal sites into legal, community- operated ones

**Table 5:** Victims of illegal mining over the last 5 years (Source: online news).

Month, Year	Location	Dead, Injured [Number of Persons]	Cause
January 2022	Atoga Timur Village, Bolaang Mongondow Timur District, North Sulawesi	2, not mentioned	poison gas
October 2021	Tumbang Torung, Kotawaringin Timur, Central Kalimantan	6, not mentioned	landslide
October 2021	Gapit Village, Sumbawa Regency, Nusa Tenggara Barat	4, not mentioned	poison gas
July 2021	Tambang Saweak, Lebong, Bengkulu	1, not mentioned	landslide
May 2021	Timbahan Nagari Abai, Solok Selatan, West Sumatera	8, 9	landslide
February 2021	Buranga Village, Parigi Moutong Regency, Central Sulawesi	6, 16	landslide
December 2020	Taman Nasional Gunung Halimun Salak (TNGHS) Lebak, Banten	4, 2 (missing)	landslide
November 2020	Sungai Seribu, Kotawaringin Barat, Central Kalimantan	10, not mentioned	landslide
October 2020	Sekatak Buji Village, Bulungan Regency, North Kalimantan	5, not mentioned	landslide
December 2019	Pulau Baru, Merangin Regency, Jambi	4, 2 (missing)	landslide
July 2019	Bakan Village, Bolaang Mongodow Regency, North Sulawesi	2, not mentioned	landslide
May 2019	Gunung Pongkor, Bogor, West Java	5, 15	landslide
March 2019	Bakan Village, Bolaang Mongodow Regency, North Sulawesi	16,18	landslide
August 2018	Gunung Botak, Wamsait Village, Buru Regency, Maluku	2, 2	landslide
June 2018	Bakan Village, Bolaang Mongodow Regency, North Sulawesi	5, not mentioned	landslide
June 2018	Gunung Suge, West Lombok	7, 6	poison gas

**Table 6:** Mercury intoxication and its effects on health.

Location	Clinical Symptoms	Ranges Mercury Concentration	Remark	Reference
Hulawa and Ilangata, North Gorontalo, Gorontalo	Bluish gums, babinski reflex, labial reflex and tremor	2.1–144.8 µg/g in hair WHO standard 1–2 mg/kg	44 miners and inhabitants got symptoms.	Arifin, Y. et al. (2015) [83]
Bolaang Mongondow, North Sulawesi	Signs of bluish discoloration of gums, rigidity and ataxia (walking or standing), alternating movements or dysdiadochokinesia, irregular eye movements or nystagmnus, Field of vision, knee jerk reflex, biceps reflex, sensory examination, tremor	0.51–79.27 µg/g in hair WHO standard 1–2 mg/kg	50 miners and inhabitants	Arifin, Y. et al. (2017) [91]
Kurun, Gunung Mas, Central Kalimantan	Easy fatigue, headache, shaking/shivering, and stiff joints	0.5178–10.4682 µg/g in hair WHO standard 1–2 mg/kg	80.5% miners got mercury contamination	Lestarisa, T. (2010) [95]
Talakiak Village, Sangir, South Solok, West Sumatera	Stiff joint disease, muscle pain, rheumatism, aches, foot/hand joints feel tingling, achy, tired, shivering/shaking, fever, sore waist and chest pain), and skin diseases of itching/itching/allergy	No data	22 miners (39%) got symptoms	Putri, G. E. (2017) [96]
Cisarua Village, Nangung, Bogor, West Java	Tremor, frequent tingling, stiff facial muscles, eye irritation, metallic taste in the mouth, muscle aches and spasms, thickened skin on the palms and soles, and headaches	0.28–68 µg/g in hair WHO standard 1–2 mg/kg	24 miners (60%) have mercury intoxication.	Junita, N. R. (2013) [97]
Lebaksitu Village, Banten	No data	0.00–188.28 μg/L in blood WHO standard (5–10 µg/L)	77.9% respondents mercury in blood more than 10 µg/L.	Kristianingsih, Y. (2018) [98]
Bulawa, Bone Bolango Regency, Gorontalo	No data	2.92–378.90 µg/L in blood Standard 8.0 µg/L 0.48–260.20 µg/g in hair Standard 2.0 µg/g	52 respondents have mercury content in the blood that exceeds the standard and 57 respondents have mercury content in their hair that exceeds the standard.	Singga, S. (2013) [99]
Kayeli Village, Gunung Botak, Buru Regency, Maluku	No data	0.10–3.25 ppm in hair Standard 0.5 ppm	Repondents are inhabitants	Rumatoras, et al. (2016) [85]

**Table 7:** Comparison between illegal ASGM and mining companies in Indonesia.

Aspect	Illegal ASGM	Mining Company
Legality	unlicensed	licensed
Number	uncountable	countable
Production	unrecorded	well recorded
Number of workers	unrecorded	recorded
Safety	uncontrolled	controlled
Health insurance	without health insurance	with health insurance
Environmental safety	unconcerned	concerned
Post-mining reclamation	no	conducted
Community safety	unconcerned	concerned
Surrounding community economic impact	direct	indirect
Social impact	high	moderate
Taxable	no	yes
Conflict	high	high

## Data Availability

No data.

## References

[1] Indonesia Gold Production. https://www.ceicdata.com/.

[2] (2021). Gold Mine Production. https://www.gold.org/goldhub/data/historical-mine-production.

[3] Prabawa F.Y. (2020). Pemodelan Sistem Dinamis Paramagnon Emas Rakyat Menuju Pertambangan Berkelanjutan, Studi Penambangan Emas rakyat Desa Kertajaya Kecamatan Simpenan Kabupaten Sukabumi Jawa Barat.

[4] Kementerian Lingkungan Hidup dan Kehutanan (2017). Grand Design, Pengurangan dan Penghapusan Merkuri Pada Pertambangan Emas Skala Kecil. http://sib3pop.menlhk.go.id/index.php/articles/view?slug=pertambangan-emas-skala-kecil-pesk.

[5] Dondo S.M., Kiyai B., Palar N. (2021). Dampak sosial pengelolaan tambang emas di Desa Bakan Kabupaten Bolaang Mongondow. J. Jar. Adm. Pembang..

[6] Redi A. (2016). Dilema Penegakan Hukum Penambangan Mineral dan Batubara Tanpa Izin pada Pertambangan Skala Kecil. J. Rechts Vinding.

[7] Nopriadi (2016). Solution on handling of illegal gold mining activities in Kuantan Singingi Region. Int. J. Appl. Environ. Sci..

[8] Krisnayanti B.D. (2018). ASGM Status in West Nusa Tenggara Province, Indonesia. J. Degrad. Min. Lands Manag..

[9] Nugroho H. (2020). Pandemi COVID-19: Tinjau ulang kebijakan mengenai PETI (Pertambangan Emas Tanpa Izin) di Indonesia. Indones. J. Dev. Plan..

[10] Perks R., Schneck N. (2021). COVID-19 in artisanal and small-scale mining communities: Preliminary results from a global rapid data collection exercise. Environ. Sci. Policy.

[11] Bansah K.J. (2019). From diurnal to nocturnal: Surviving in a chaotic artisanal and small-scale mining sector. Resour. Policy.

[12] Aisyah Syafei A. (2019). Penyediaan Alternatif Teknologi Pengolahan Emas Non Merkuri. https://sitkb3.menlhk.go.id/infomerkuri/?p=4652.

[13] Nafsiatun, Saptomo P., Najib W., Hartini (2019). Characteristics of Environmental Conflicts Caused by Illegal Gold Mining in West Kalimantan, Indonesia. J. Humanit. Soc. Sci..

[14] Trimiska L., Wiryono W., Suhartoyo H. (2018). Kajian Penambangan Emas Tanpa Izin (PETI) di Kecamatan Lebong Utara, Kabupaten Lebong. Naturalis.

[15] Herman D.Z. Pertambangan Tanpa Izin (PETI) dan Kemungkinan Alih Status Menjadi Pertambangan Skala Kecil. Buletin Sumber Daya Geologi. http://psdg.geologi.esdm.go.id/buletin_pdf_file.

[16] Darmono D., Darmono D. (2009). Mineral dan Energi Kekayaan Bangsa: Sejarah Pertambangan dan Energi Indonesia.

[17] Rahmana S. (2018). Pengaruh Pendirian Perusahaan Pertambangan Emas Kolonial Belanda di Lebong Tahun 1897–1930. J. Aghinya Stiesnu Bengkulu.

[18] Mulyadi I., Zaman B., Sumiyati S. (2020). Mercury Concentrations of River Water and Sediment in Tambang Sawah Village Due to Unlicensed Gold Mining. J. Ilm. Tek. Kim..

[19] Buchori M.I.E. (2019). Kerusakan Lahan Akibat Kegiatan Penambangan Emas Tanpa Izin di Sekitar Sungai Singingi Kabupaten Kuantan Singingi. J. Pembang. Wil. Dan Kota.

[20] Rahim S. (2017). Konflik Pemanfaatan Ruang Akibat Penambangan Emas Tanpa Ijin (PETI) di Kawasan Hutan Produksi Terbatas. J. GeoEco.

[21] Andriyanto R., Fitrisia A. (2019). Eksplorasi dan eksploitasi penambangan emas Lebong Donok (Bengkulu) tahun 1897–1942. Kronologi.

[22] https://peraturan.bpk.go.id/Home/Details/53563/perpu-no-37-tahun-1960.

[23] Carlile J.C., Mitchell A.H.G. (1994). Magmatic Arcs and Associated Gold and Copper Mineralization in Indonesia. J. Geochem. Explor..

[24] https://peraturan.bpk.go.

[25] Male Y.T., Reichelt-Brushett A.J., Pocock M., Nanlohy A. (2013). Recent mercury contamination from artisanal gold mining on Buru Island, Indonesia—Potential future risks to environmental health and food safety. Mar. Pollut. Bull..

[26] Mariwy A., Male Y.T., Manuhutu J.B. (2019). Mercury (Hg) Contents Analysis in Sediments at Some River Estuaries in Kayeli Bay Buru Island. IOP Conference Series: Materials Science and Engineering.

[27] Agus A., Dwimawanti I.H. Rationality Conflict Between the Government and The Community: A Case Study on Illegal Gold Mining in Prabu Village, West Nusa Tenggara Province. Proceedings of the 4th International Conference on Indonesian Social and Political Enquiries.

[28] Law of the Republic of Indonesia Number 4 of 2009 on Mineral and Coal Mining. http://www.apbi-icma.org/uploads/files/old/2013/11/uu_no_4_2009_en.pdf.

[29] https://peraturan.bpk.go.id/Home/Details/138909/uu-no-3-tahun-2020.

[30] Aziz M. (2014). The model of traditional gold mining and its environmental management in the Paningkaban Village, Gumelar District, Banyumas Regency, Central Java. Din. Rekayasa.

[31] Kementerian Energi dan Sumber Daya Mineral (2021). Perkembangan Kebijakan Sub Sektor Pertambangan Mineral dan Batubara. http://www.minerba.esdm.go.id.

[32] Suherman A. (2012). Usaha Tambang Emas. https://www.academia.edu/6849960/USAHA_TAMBANG_EMAS.

[33] Zulkarnain I. (2006). Mengenal Fenomena PETI di Kawasan Pertambangan Emas Pongkor. http://lipi.go.id/berita/mengenal-fenomena-peti-di-kawasan-pertambangan-emas-pongkor/233LIPI.

[34] Hasibuan O., Tjakraatmadja J.H., Sunitiyoso Y. (2021). Finding workable and mutually beneficial solution to eradicate illegal gold mining. Bisnis Birokrasi J. Ilmu Adm. Dan Organ..

[35] Kasworo Y. (2015). Pertambangan Emas Tanpa Izin (PETI), Dapatkah Ditanggulangi?. J. Rechtsvinding.

[36] Nuzul A.Y. (2018). Dampak Pertambangan Emas Ilegal di Aliran Sungai Batanghari Kabupaten Dharmasraya Sumatera Barat. Res. Gate.

[37] Santoso D.H., Gomareuzzaman M. (2018). Kelayakan teknis penambang emas pada wilayah pertambangan rakyat (Studi kasus: Desa Kalirejo, Kecamatan Kokap, Kabupaten Kulon Progo. J. Sci. Technol..

[38] Sittadewi E.H. (2016). Mitigasi lahan terdegradasi akibat penambangan melalui revegetasi. J. Sains Dan Teknol. Mitigasi Bencana.

[39] Puluhulawa U.F., Harun A.A. (2019). Biodiversity protection from the impact of illegal gold mining for sustainability. IOP Conference Series: Earth and Environmental Science.

[40] Bruno E.D., Rubane D.A., Tiess G., Perrone B., Perrota P., Mikhailenko A., Ermolev V., Yashalova N. (2019). Artisanal and small-scale gold mining, meandering tropical rivers, and geological heritage: Evidence from Brazil and Indonesia. Sci. Total Environ..

[41] Amelia N.R., Kartodihardjo H., Sundawati L. (2019). Peran modal sosial masyarakat penambang emas dalam mempertahankan tambang ilegal di Taman Hutan Raya Sulawesi Tengah. J. Sylva Lestari.

[42] Wawo A.R.H., Widodo S., Jafar N., Yusuf F.N. (2017). Analisis pengaruh penambangan emas terhadap kondisi tanah pada pertambangan rakyat Poboya Palu, Provinsi Sulawesi Tengah. J. Geomine.

[43] Basuki A. (2018). Penegakan Hukum Terhadap Tindak Pidana Penambangan Tanpa Izin oleh Polres Landak (Tinjauan Yuridis-Sosiologis). J. Nestor Magister Huk..

[44] Erb M., Mucek A.E., Robinson K. (2021). Exploring a social geology approach in eastern Indonesia: What are mining territories?. Extr. Ind. Soc..

[45] Khotami (2020). Institution building dalam mengatasi persoalan pertambangan emas tanpa izin di Kabupaten Kuantan Singingi Provinsi Riau. Nakhoda.

[46] Triyana I., Ikhwan I. (2019). Dinamika Sosial dan Ekonomi Pekerja Tambang Emas Pasca Ditutupnya Tambang Emas Ilegal di Nagari Palangki Kabupaten Sijunjung. Cult. Soc. J. Anthropol. Res..

[47] Susanti T., Hidayat, Sartika D., Utami W., Viktres R.H., Novallyan D. (2019). The influence of illegal gold mining (IGM) on environmental, economic, and educational sectors of Muara Mensao Village, Jambi. Int. Conf. Basic Sci. Its Appl..

[48] Solichin E. (2020). Implications of Illegal Gold Mining on the Household Economy and the Environment. Saudi J. Bus. Manag. Stud..

[49] Hasibuan O.P., Tjakraatmadja J.H., Sunitiyoso Y. (2020). Illegal gold mining in Indonesia: Structure and causes. Int. J. Emerg. Mark..

[50] Rosjadi D., Taufiq M. (2019). Efektivitas peranan kepolisian dalam menertibkan penambangan emas tanpa izin (PETI) yang dilakukan oleh masyarakat di lahan penambangan PT AntamTbk dari sisi pembangunan berkelanjutan. J. Huk. De’rechtsstaat.

[51] Pamungkas P.D. (2018). Efektivitas penyidikan tindak pidana penambangan emas tanpa izin di Kabupaten Solok Selatan (Studi pada Direktorat Reserse Kriminal Khusus Kepolisian Daerah Sumatera Barat). UNES Law Rev..

[52] Arif I. (2020). Emas Indonesia.

[53] Ismawati Y. (2014). Gold, Mercury, and the Next Minamata. Indones. J. Leadersh. Policy World Aff..

[54] Guswahyuni S.M. Pertambangan Emas Tanpa Izin dan Masalah Public Health Dari Anak-Anak Yang Tinggal di Sekitar. Proceedings of the 3rd UGM Public Health Symposium.

[55] Bank M.S. (2020). The mercury science-policy interface: History, evolution and progress of the Minamata Convention. Sci. Total Environ..

[56] Krisnayanti B.D., Probiyantono A.S. (2020). Teknologi Pengolahan Emas Pada Pertambangan Emas Skala Kecil di Indonesia, buku 4.

[57] Mahmud M. (2012). Model Sebaran Spasial Temporal Konsentrasi Merkuri Akibat Penambangan Emas Tradisional Sebagai Dasar Monitoring dan Evaluasi Pencemaran di Ekosistem Sungai Tulabolo Provinsi Gorontalo. Ph.D. Thesis.

[58] Gundo S.D.I., Polii B.J.V., Umboh J.M.L. (2020). Kandungan merkuri pada penambang emas rakyat. Indones. J. Public Health Community Med..

[59] Prasetia H., Sakakibara M., Omori K., Laird J.S., Sera K., Kurniawan I.A. (2018). Mangifera indica as bioindicator of mercury atmospheric contamination in an ASGM area in North Gorontalo Regency, Indonesia. Geosciences.

[60] Zhang H., Feng X., Larssen T., Qiu G., Vogt R.D. (2010). In inland China, rice, rather than fish, is the major pathway for methylmercury exposure. Env. Health Perspect.

[61] Junaidi M., Krisnayanti B.D., Juharfa J., Anderson C. (2019). Risk of Mercury Exposure from Fish Consumption at Artisanal Small-Scale Gold Mining Areas in West Nusa Tenggara, Indonesia. J. Health Pollut..

[62] Ekawanti A., Krisnayanti B.D. (2015). Effect of Mercury Exposure on Renal Function and Hematological Parameters among Artisanal and Small-scale Gold Miners at Sekotong, West Lombok, Indonesia. Health Pollut..

[63] Ismawati Y. (2016). Children’s Exposure to Mercury in Artisanal and Small-Scale GOLD Mining Areas in Indonesia and in More than 70 Countries.

[64] Afrifa J., Opoku Y.K., Gyamerah E.O., Ashiagbor G., Sorkpor R.D. (2019). The clinical importance of the mercury problem in artisanal small-scale gold mining. Front. Public Health.

[65] Undang-Undang Nomor 11 tahun 2017 Tentang Pengesahan Minamata Convention on Mercury. https://peraturan.bpk.go.id/Home/Details/53614#:~:text=UU%20No.%2011%20Tahun%202017,Merkuri).

[66] Lembaran Negara Republik Indonesia. https://peraturan.go.id/common/dokumen/ln/2019/ps21-2019.pdf.

[67] Kementerian Energi dan Sumber Daya Mineral. 2021. Perkembangan Kebijakan Sub Sektor Pertambangan Mineral dan Batubara. https://jdih.esdm.go.id/storage/document/Permen%20ESDM%20Nomor%2016%20Tahun%202020.pdf.

[68] Djatmiko A., Purwendah E.K., Pudyastiwi E. (2019). Benefits of Indonesia Ratification of Minamata Convention on Mercury. Int. J. Bus. Econ. Law.

[69] https://www.goldismia.org/sites/default/files/2021-06/Annual%20Report%202020-8.pdf.

[70] Spiegel S.J., Agrawal S., Mikha D., Vitamerry K., Billon P.L., Veiga M., Konolius K., Paul B. (2018). Phasing Out Mercury? Ecological Economics and Indonesia’s Small-Scale Gold Mining Sector. Ecol. Econ..

[71] Aylmore M.G., Muir D.M. (2001). Thiosulfate leaching of gold- A. Review. Miner. Eng..

[72] Yustanti E., Guntara A., Wahyudi T. (2018). Ekstraksi Bijih Emas Sulfida Tatelu Minahasa Utara Menggunakan Reagen Ramah Lingkungan Tiosulfat. Teknika.

[73] Wahyudin P., Mubarok M.Z. (2016). Perilaku Adsorpsi Emas dari Larutan Ammonium Thiosulfat dengan Karbon Aktif dan Resin Penukar Ion. Metalurgi.

[74] Matsumoto Y., Kasamatsu H., Sakakibara M. (2022). Challenges in Forming Transdisciplinary Communities of Practice for Solving Environmental Problems in Developing Countries. World Futures.

[75] Hindratmo B., Rita R., Masitoh S., Kusumardhani M., Junaedi E. (2019). Kandungan Logam Berat Merkuri (Hg) pada Area Bekas Penambangan Emas Skala Kecil (PESK): Studi Kasus di Gunung Botak, Kabupaten Buru, Provinsi Maluku. Ecolab.

[76] Indah M.F., Agustina N., Ariyanto E. (2020). Analysis of Mercury Levels, Degree of Acidity and Health Risk Factors for Unlicensed Gold Miners in Cempaka Sub-District. Bul. Penelit. Kesehat..

[77] Palapa T.M., Maramis A.A. (2015). Heavy Metals in Water of Stream Near an Amalgamation Tailing Ponds in Talawaan –Tatelu Gold Mining, North Sulawesi, Indonesia. Procedia Chem..

[78] Hidayanti K. (2019). Distribusi logam berat pada air dan sedimen serta potensi bioakumulasi pada ikan akibat penambangan emas tanpa izin (Studi kasus: DAS Sekonyer, Kalimantan Tengah). Media Ilm. Tek. Lingkung..

[79] Fakaubun F.R., Male Y.T., Selanno D.A.J. (2020). Bioconcentration and Bioaccumulation of Mercury (Hg) in Seagrass *Enhalus Acoroides* in Kayeli Bay, Buru Regency, Maluku Province. Indones. J. Chem. Res..

[80] Susanti T., Utami W., Hidayat H. (2018). The negative impact of illegal gold mining on the environmental sector in Batang Asai, Jambi. Sustinere J. Environ. Sustain..

[81] Sakakibara M., Sera K., Kurniawan I.A. (2017). Mercury contamination of cattle in artisanal and small-scale gold mining in bombana, Southeast Sulawesi, Indonesia. Geosciences.

[82] Harianja A.H., Saragih G.S., Fauzi R., Hidayat M.Y., Syofyan Y., Tapriziah E.R., Kartiningsih S.E. (2020). Mercury Exposure in Artisanal and Small-Scale Gold Mining Communities in Sukabumi, Indonesia. J. Health Pollut..

[83] Arifin Y., Sakakibara M., Sera K. (2015). Impacts of artisanal and Small-Scale Gold Mining (ASGM) on environment and human health of Gorontalo Utara Regency, Gorontalo Province, Indonesia. Geosciences.

[84] Sakakibara M., Sera K. (2017). Current mercury exposure from artisanal and small-scale gold mining in Bombana, Southeast Sulawesi, Indonesia—future significant health risks. Toxics.

[85] Rumatoras H., Taipabu M.I., Lesiela L. (2016). Analysis of mercury (Hg) content on hair villagers Kayeli, ilegal gold mining result in Botak Mountain Area, Buru Regency-Maluku Province. Ind. J. Chem. Res..

[86] Planet Gold Gold-Ismia. https://www.planetgold.org/indonesia.

[87] (2019). Program Emas Rakyat Sejahtera. Quaterly Email Newsletters. https://pers.no-hg.org/wordpress/wp-content/uploads/2019/12/e-Newsletter-PERS_Bahasa-Version_Final_Final.pdf.

[88] Zuhdi S., Wahyudi B., Munawwaroh T. (2018). The role local goverment in gold mine conflict handling in Trenggalek Regency, East Jawa Province. J. Prodi Damai Dan Resolusi Konflik.

[89] Libassi M. (2020). Matthew Libassi Mining heterogeneity: Diverse labor arrangements in an Indonesian informal gold economy. Extr. Ind. Soc..

[90] Abbas H.H., Sakakibara M., Sera K., Arma L.H. (2017). Mercury Exposure and Health Problems in Urban Artisanal Gold Mining (UAGM) in Makassar, South Sulawesi, Indonesia. Geosciences.

[91] Arifin Y.I., Sakakibara M., Sera K. (2017). Heavy metals concentrations in scalp hairs of ASGM miners and inhabitants of the Gorontalo Utara regency. IOP Conf. Ser. Earth Environ. Sci..

[92] Zaharani F. (2015). Salami IRS. Kandungan Merkuri Pada Urin Dan Rambut Sebagai Indikasi Paparan Merkuri Terhadap Pekerja Tambang Emas Tanpa Izin (Peti) Di Desa Pasar Terusan Kecamatan Muara Bulian Kabupaten Batanghari–Jambi. J. Teh Lingkung.

[93] Sumantri A., Laelasari E., Junita N.R., Nasrudin N. (2014). Logam Merkuri pada Pekerja Penambangan Emas Tanpa Izin. Kesmas Natl. Public Health J..

[94] Ismawati Y. (2015). Preliminary Environmental Health Report from 3 ASGM Hotspots in Indonesia: Bombana, Sekotong and Cisitu. https://www.academia.edu/16013226/Preliminary_environmental_health_report_from_3_ASGM_Hotspots_in_Indonesia_Bombana_Sekotong_and_Cisitu.

[95] Lestarisa T. (2010). Faktor-Faktor Yang Berhubungan Dengan Keracunan Merkuri (Hg) Pada Penambang Emas Tanpa Ijin (PETI) di Kecamatan Kurun, Kabupaten Gunung Mas, Kalimantan Tengah. Master’s Thesis.

[96] Putri G.E. (2017). Gejala Kesehatan yang diderita penambang emas akibat proses penambangan emas menggunakan merkuri (Hg). Med. St..

[97] Junita N.R. (2013). Resiko keracunan merkuri (Hg) pada pekerja penambang emas tanpa izin (PETI) di Desa Cisarua Kecamatan Nanggung Kabupaten Bogor tahun 2013. Bachelor Thesis.

[98] Kristianingsih Y. (2018). Bahaya merkuri pada masyarakat di Pertambangan Emas Skala Kecil (PESK) Lebaksitu. J. Ilm. Kesehat..

[99] Singga S. (2013). Analisis resiko kesehatan pajanan pada masyarakata Kecamatan Bulawa, Kabupaten Bone Bolango Provinsi Gorontalo. J. MKMI.

[100] https://jdih.esdm.go.id/storage/document/PP%20No.%2023%20Thn%202010.pdf.

[101] https://jdih.esdm.go.id/storage/document/UU%2032%20Tahun%202009%20(PPLH).pdf.

[102] https://peraturan.bpk.go.id/Home/Details/4835/pp-no-23-tahun-2008#:~:text=PP%20No.%2023%20Tahun%202008,Penanggulangan%20Bencana%20%5BJDIH%20BPK%20RI%5D.

[103] https://peraturan.bpk.go.id/home/Details/38685/uu-no-23-tahun-2014.

[104] Muslihudin M., Santosa I., Setyoko P.I., Bahtiar R.A. (2020). Local Government’s Role and Policy on Illegal Mining (case Study of gold Mining in Banyumas Indonesia). Am. J. Humanit. Soc. Sci. Res..

[105] Muryani E. (2019). Sinergisitas Penegakan Hukum Pada Kasus Pertambangan Emas Tanpa Izin di Kabupaten Banyumas, Jawa Tengah. J. Best..

[106] Nainggolan P. (2018). Resistensi Penambang Ilegal: Studi Kasus eksploitasi Tambang Galian B (Emas) di Desa Sayurmatua Kecamatan Naga Juang Kabupaten Mandailing Natal. J. Buana.

[107] Yani R.F., Asrinaldi A., Rahmadi D. (2019). Peran WALHI Sumbar dalam Investigasi Tambang Emas Ilegal di Kota Padang. J. Demokr. Dan Polit. Lokal.

[108] Damaryanti H., Yenny A.S. (2020). Implementation of Customary Sanction “Pengocek Torun” in Dayak Simpakng Community to Settlement of Illegal Gold Mining Ketapang Regency. Adv. Soc. Sci. Educ. Humanit. Res..

[109] Tinov M.T. (2019). Strengthening institutions in the effort adat costumary law enforcement in illegal gold mining areas affected. J. Niara.

[110] Pateda S. (2021). Personal communication.

[111] Krisnayanti B.D., Probiyantono A.S. (2020). Penggunaan merkuri dan dampaknya terhadap lingkungan serta sebaran lokasi pertambangan emas skala kecil, buku 2. https://www.goldismia.org/sites/default/files/2021-01/View%20Buku%202.pdf.

[112] Wafid M.A.N. (2022). Personal communication.

